# Post-pandemic resurgence and collapse of *Mycoplasma pneumoniae* in hospitalized children: a four-year multiplex surveillance study in China

**DOI:** 10.3389/fpubh.2026.1833309

**Published:** 2026-06-04

**Authors:** Hongbo Hu

**Affiliations:** Department of Clinical Laboratory, Maternal and Child Health Hospital of Hubei Province, Wuhan, China

**Keywords:** multiplex PCR surveillance, *Mycoplasma pneumoniae*, pediatric pneumonia, post-pandemic epidemiology, respiratory pathogen ecology

## Abstract

**Background:**

Understanding the post-pandemic behavior of *Mycoplasma pneumoniae* (MP) in children is important for respiratory pathogen surveillance and public health preparedness. This study characterized the full epidemic cycle of MP after the relaxation of COVID-19 restrictions and examined its ecological context and associated pneumonia risk.

**Methods:**

Four years of multiplex PCR testing data (2022–2025) from a provincial maternal and child health hospital in China were analyzed. Temporal trends in MP activity were assessed using generalized additive models. Monthly activity of co-circulating respiratory pathogens-including respiratory syncytial virus (RSV), influenza A virus (FluA), and human rhinovirus (HRV)-was compared using standardized positivity and ecological correlations. Pneumonia risk among MP-infected children was evaluated with multivariable logistic regression.

**Results:**

*Mycoplasma pneumoniae* showed a pronounced boom-and-bust cycle, with low activity in 2022, a sharp resurgence in 2023, and near-disappearance by 2025. As MP circulation declined, RSV, FluA, and HRV showed increased activity, indicating dynamic shifts within the respiratory pathogen ecosystem. Pneumonia risk was highest during the 2023 resurgence, remained elevated in 2024, and was no longer significantly elevated by 2025.

**Conclusion:**

This study documents a complete post-pandemic MP epidemic cycle in hospitalized children in Hubei Province. The observed pattern is compatible with hypotheses of immunity debt and subsequent susceptible depletion, although direct measurement of these mechanisms was beyond the scope of this study. These findings highlight the need for continuous multi-pathogen surveillance and clinical preparedness during MP resurgence waves, as elevated pneumonia risk may extend beyond peak transmission periods.

## 1 Introduction

*Mycoplasma pneumoniae* (MP) is a major cause of community-acquired pneumonia in children. Its circulation has historically followed characteristic multi-year epidemic cycles shaped by fluctuating population immunity, transmission dynamics, and periodic shifts in circulating genotypes (1, 2). Before the COVID-19 pandemic, MP epidemics occurred every 3–7 years in many countries and were associated with substantial clinical and public health burden (3–5). During the pandemic, widespread non-pharmaceutical interventions (NPIs) led to an unprecedented global suppression of MP circulation, with near-complete disappearance reported across multiple regions from 2020 to 2022 (6–8). As NPIs were relaxed in late 2022, several respiratory pathogens rebounded rapidly (9–12). MP, in contrast, showed a delayed and geographically heterogeneous resurgence beginning in 2023, prompting renewed interest in its post-pandemic epidemiology (13–15). These shifts highlight the need to understand MP dynamics within the broader context of respiratory pathogen circulation and public health preparedness, including concepts such as immunity debt (the accumulation of susceptible individuals due to prolonged low pathogen exposure) and susceptible depletion (the reduction of susceptible individuals following a large outbreak).

In China, MP circulation remained low through 2022, followed by a marked resurgence in 2023 documented in multiple regions, including the present study population (7, 16). These observations raised concerns regarding potential shifts in transmission patterns, age distribution, and clinical severity after a prolonged period of minimal exposure. However, comprehensive multi-year data describing MP activity after the lifting of COVID-19 restrictions have remained limited. Few studies examined MP trends alongside other respiratory pathogens within a standardized multiplex testing framework, leaving the broader ecological context of MP resurgence insufficiently characterized. Moreover, whether the post-pandemic rebound of MP was accompanied by changes in pneumonia risk among hospitalized children had not been systematically evaluated. Addressing these gaps is essential for informing multi-pathogen surveillance, early-warning systems, and public health planning in the post-pandemic era.

Using 4 years of multiplex respiratory pathogen testing data from a large provincial maternal and child health hospital in China (2022–2025), this study aimed to characterize: (1) the temporal dynamics of MP resurgence and decline using offset-adjusted models; (2) the ecological relationship between MP and other respiratory pathogens through *z*-score–standardized activity profiles and correlation analyses; and (3) year-to-year variation in pneumonia risk among hospitalized children with MP infection via multivariable logistic regression. Together, these analyses provide a comprehensive picture of MP dynamics in the post-COVID-19 era and clarify its ecological and clinical relationships with co-circulating respiratory pathogens in hospitalized children. Understanding these patterns is important for strengthening respiratory pathogen surveillance and guiding clinical and public health preparedness as epidemic cycles re-emerge in the post-pandemic period.

## 2 Methods

### 2.1 Study design and population

This retrospective study was conducted at a provincial tertiary maternal and child health hospital in China. All hospitalized children aged 1 month to under 18 years who underwent multiplex respiratory pathogen testing between January 2022 and December 2025 were included. Neonates younger than 1 month were not included, as they constitute a distinct immuno-epidemiologic subgroup whose infection patterns are strongly influenced by maternal antibodies, perinatal factors, and immune immaturity, and their inclusion would introduce heterogeneity unrelated to pathogen circulation or transmission dynamics. To ensure methodological consistency, only tests performed using the same commercial multiplex PCR capillary electrophoresis assay were analyzed. When multiple tests were performed during the same hospitalization, only the first result was retained. Mixed infections involving pathogens outside the multiplex panel, including bacterial pathogens, fungal pathogens, and SARS-CoV-2, were excluded to ensure that analyses were restricted to co-detections within the standardized respiratory pathogen panel. The study was approved by the Institutional Ethics Committee of the Maternal and Child Health Hospital of Hubei Province (Approval No.: 2024IEC040). All data were de-identified prior to analysis, and the requirement for informed consent was waived due to the retrospective nature of the study.

### 2.2 Respiratory pathogen testing

Nasopharyngeal swabs or bronchoalveolar lavage fluid were collected at admission following routine clinical procedures, depending on the patient’s clinical condition and sampling feasibility. A commercial multiplex PCR capillary electrophoresis panel was used to detect influenza A virus (FluA), influenza B virus (FluB), respiratory syncytial virus (RSV), human parainfluenza viruses (HPIV), human rhinovirus (HRV), human metapneumovirus (hMPV), human coronaviruses (HCoVs; NL63, OC43, 229E, and HKU1), human adenovirus (HAdV), human bocavirus (HBoV), MP, and *Chlamydia pneumoniae* (CP). All pathogens detected by the multiplex panel were included in descriptive analyses, temporal trend analyses, and ecological correlation analyses to ensure consistent evaluation of all in-panel respiratory pathogens within the same standardized testing framework.

### 2.3 Statistical analysis

All statistical analyses were performed using R version 4.5.2. Descriptive statistics were first used to summarize monthly and annual testing volumes and positivity rates, after which specific analytical models were applied to evaluate temporal trends, pathogen activity, ecological relationships, and clinical outcomes. Monthly MP positivity was calculated as the number of MP-positive tests divided by the total number of multiplex tests performed each month.

Temporal trends were modeled using a generalized additive model with a negative binomial distribution and a log link function. The model incorporated a thin-plate regression spline for time expressed as month, and the basis dimension was restricted to four to avoid overfitting. An offset term of the logarithm of total monthly tests was included to adjust for variation in testing intensity, and effective degrees of freedom with corresponding *p*-values were used to evaluate the presence of non-linear temporal patterns.

Monthly positivity rates for each pathogen were standardized using *z*-score normalization to allow comparison of seasonal activity across pathogens with different baseline prevalence. Ecological relationships among pathogens were assessed using Spearman correlation coefficients calculated from monthly aggregated positivity rates.

Among hospitalized children with MP infection, pneumonia was defined based on clinical diagnosis supported by radiographic evidence. Multivariable logistic regression was used to estimate adjusted odds ratios for pneumonia by calendar year, adjusting for age in months as a continuous variable, sex, and infection type defined as MP single infection versus co-infection with other respiratory pathogens. Ninety-five percent confidence intervals were reported.

A sensitivity analysis was performed by re-evaluating MP temporal trends using a negative binomial regression model with year treated as a categorical predictor and the logarithm of total monthly tests included as the offset. This model did not assume a non-linear smooth function of time and therefore provided a complementary assessment of annual differences in MP activity. Statistical significance was defined as a two-sided *p*-value less than 0.05.

## 3 Results

### 3.1 Annual testing volume and overall positivity of respiratory pathogens, 2022–2025

Annual testing volume declined from 12,560 in 2022 to 4,334 in 2025 (Table 1). *Mycoplasma pneumoniae* positivity showed substantial year-to-year variation, rising from 5.7% in 2022 to 17.8% in 2023, falling to 7.9% in 2024, and reaching only 0.4% in 2025. The study population consisted of 30,966 hospitalized children (57.6% male, 42.4% female), with a median age ranging from 4.8 to 6.7 years across the study period.

**Table 1:** Annual testing volume and positivity of respiratory pathogens (2022–2025).

Year	Total tests	MP, *n* (%)	Age, median (IQR), years	RSV, *n* (%)	FluA, *n* (%)	HRV, *n* (%)	HAdV, *n* (%)	hMPV, *n* (%)	HPIV, *n* (%)	HBoV, *n* (%)	FluB, *n* (%)	HCoV, *n* (%)	CP, *n* (%)
2022	12,560	721 (5.74)	5.9 (3.3–7.7)	655 (5.21)	392 (3.12)	1,898 (15.11)	797 (6.35)	1,115 (8.88)	735 (5.85)	193 (1.54)	387 (3.08)	185 (1.47)	18 (0.14)
2023	9,087	1,621 (17.84)	6.7 (4.4–8.4)	969 (10.66)	800 (8.80)	1,747 (19.23)	268 (2.95)	577 (6.35)	598 (6.58)	136 (1.50)	42 (0.46)	140 (1.54)	14 (0.15)
2024	4,985	394 (7.90)	4.8 (2.9–7.1)	248 (4.97)	293 (5.88)	1,177 (23.61)	608 (12.20)	216 (4.33)	268 (5.38)	68 (1.36)	132 (2.65)	135 (2.71)	37 (0.74)
2025	4,334	17 (0.39)	5.9 (4.3–7.8)	582 (13.43)	360 (8.31)	891 (20.56)	134 (3.09)	252 (5.81)	247 (5.70)	51 (1.18)	4 (0.09)	60 (1.38)	50 (1.15)

MP, *Mycoplasma pneumoniae*; RSV, respiratory syncytial virus; FluA, influenza A virus; FluB, influenza B virus; HRV, human rhinovirus; HAdV, human adenovirus; hMPV, human metapneumovirus; HPIV, human parainfluenza virus; HBoV, human bocavirus; HCoV, human coronaviruses; CP, *Chlamydia pneumoniae*.

Other respiratory pathogens also exhibited marked annual fluctuations. Respiratory syncytial virus and influenza A virus reached their highest annual positivity in 2023 (10.7 and 8.8%, respectively). Human rhinovirus remained prominent throughout the study period, with the highest proportions observed in 2024 (23.6%) and 2025 (20.6%). Human adenovirus and human metapneumovirus showed intermediate but variable activity, whereas HPIV, HBoV, FluB, HCoV, and CP accounted for smaller proportions each year.

Monthly MP positivity in Supplementary Figure 1 demonstrates low activity throughout 2022, a discrete mid-2023 surge, and a sustained decline during 2024–2025, consistent with the annual patterns summarized in Table 1.

### 3.2 Temporal dynamics of *Mycoplasma pneumoniae*, 2022–2025

**Figure 1:**
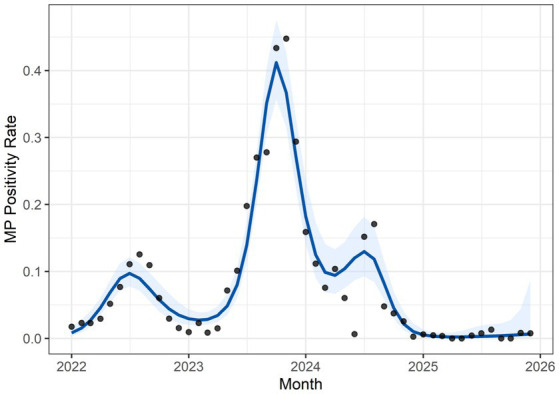
Temporal dynamics of *Mycoplasma pneumoniae* positivity, 2022–2025. Monthly *M. pneumoniae* positivity based on all MP-positive detections, including both single and mixed infections. Black dots represent observed monthly positivity. The blue line shows the fitted trend from a negative-binomial generalized additive model with an offset for monthly testing volume. Shaded areas indicate 95% confidence intervals.

Monthly GAM-smoothed estimates revealed a pronounced boom-and-bust pattern in MP activity from 2022 to 2025 (Figure 1). MP positivity increased steadily during 2022, rising from approximately 0.8% in January to a mid-year peak of around 9.7%, before declining to below 3.5% by December. After a brief low in early 2023, MP activity surged sharply from May onward, reaching a fitted peak of 35.1% in September 2023 (observed peak: 44.8% in November 2023). MP positivity then declined throughout 2024, stabilizing at approximately 1.0–2.7% by late 2024, and remained at near-zero levels across 2025, with several months recording zero observed positives.

To quantify annual changes and assess robustness, a negative binomial sensitivity analysis was performed using half-year aggregates (Supplementary Table S1). Compared with the first half of 2023 (2023H1), MP incidence was significantly higher in 2022H2 (IRR 2.34, 95% CI 1.94–2.83) and peaked in 2023H2 (IRR 7.64, 95% CI 6.46–9.11). Incidence declined in 2024H1 (IRR 2.20, 95% CI 1.71–2.81) and 2024H2 (IRR 1.64, 95% CI 1.32–2.04), followed by a marked collapse in 2025H1 (IRR 0.085, 95% CI 0.038–0.161) and persistently minimal activity in 2025H2 (IRR 0.135, 95% CI 0.053–0.279). These findings confirm the sharp rise in late 2023 and the near-disappearance of MP circulation throughout 2025.

### 3.3 Temporal patterns of respiratory pathogens, 2022–2025

**Figure 2:**
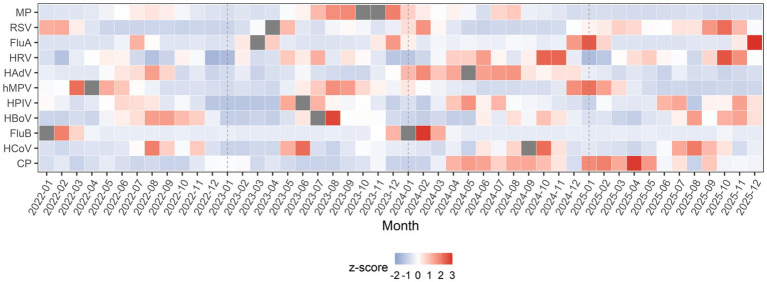
Seasonal activity patterns of respiratory pathogens based on *z*-score–standardized monthly positivity, 2022–2025. Monthly positivity for each pathogen was standardized using *z*-score normalization to allow comparison of seasonal activity across pathogens with different baseline prevalence. Higher *z*-scores indicate months with above-average activity relative to each pathogen’s own distribution.

Monthly *z*-score–standardized positivity revealed distinct temporal patterns across respiratory pathogens from 2022 to 2025 (Figure 2). *Mycoplasma pneumoniae* showed a pronounced elevation in mid-2023, with z-scores exceeding 3 during September–November 2023, followed by a rapid decline throughout 2024–2025. Respiratory syncytial virus displayed intermittent increases, including clear elevations in early 2023 and early 2024–2025. Influenza A virus exhibited several sharp, short-lived peaks, most prominently in March 2023 and December 2024–January 2025. Human rhinovirus demonstrated recurrent positive deviations across multiple years, with higher *z*-scores in mid-2023 and late 2024. Human adenovirus and human metapneumovirus showed heterogeneous fluctuations with episodic high-*z*-score months, whereas HPIV and HBoV exhibited repeated positive deviations. Influenza B virus and human coronaviruses showed more modest but variable increases.

The correlation matrix of monthly positivity (Supplementary Figure 2) showed both positive and negative correlations of varying strength. Most correlations fell within the weak (|*ρ*| < 0.3) to moderate (0.3 ≤ |ρ| < 0.6) range. Moderate positive correlations were observed for MP with HAdV (*ρ* = 0.37) and hMPV (*ρ* = 0.38), for HRV with HPIV (*ρ* = 0.51), and for HBoV with HCoV (*ρ* = 0.56). Moderate negative correlations were identified for MP with RSV (*ρ* = −0.37) and *Chlamydia* spp. (*ρ* = −0.48). These values reflect weak-to-moderate temporal co-occurrence or inverse patterns among pathogens over the four-year period, highlighting both the direction and the magnitude of their associations.

### 3.4 Pneumonia risk among hospitalized children with MP infection, 2022–2025

**Figure 3:**
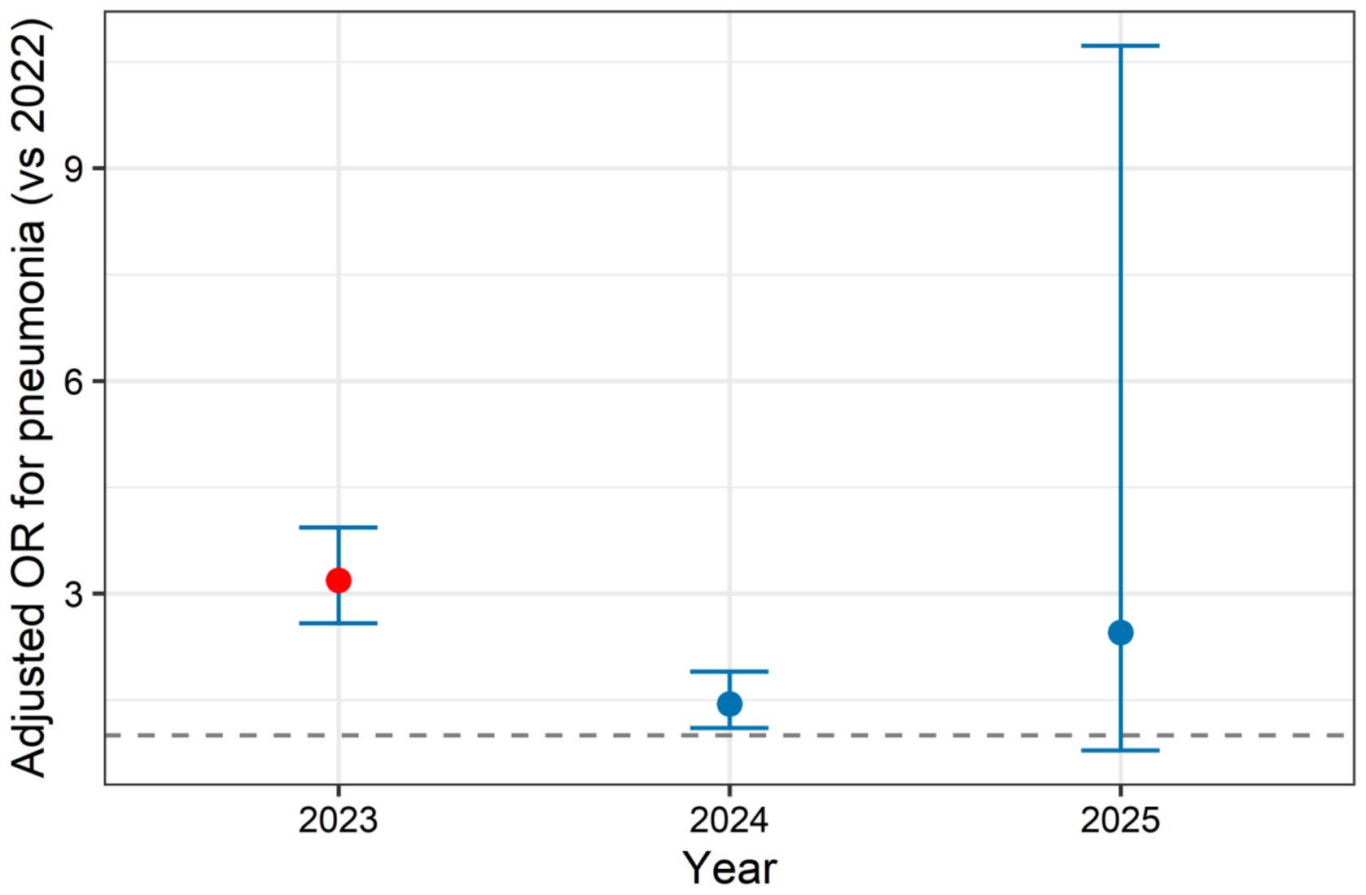
Adjusted odds of pneumonia among children with *M. pneumoniae* infection, 2022–2025. Adjusted odds ratios for pneumonia were estimated using multivariable logistic regression, adjusting for age, sex, and infection type (MP single infection vs. co-infection). Error bars represent 95% confidence intervals.

Multivariable logistic regression showed substantial year-to-year variation in pneumonia risk among hospitalized children with MP infection (Figure 3). Risk was highest in 2023, remained elevated in 2024, and returned to baseline by 2025. Compared with 2022, the adjusted odds of pneumonia were significantly higher in 2023 (OR, 3.19; 95% CI, 2.58–3.94; *p* < 0.001) and 2024 (OR, 1.45; 95% CI, 1.10–1.90; *p* = 0.008). In 2025, no significant difference was observed (OR, 2.45; 95% CI, 0.79–10.73; *p* = 0.162), with wide confidence intervals reflecting the small number of MP-positive cases that year.

## 4 Discussion

This four-year study, spanning the period immediately after the lifting of COVID-19 restrictions, provides a comprehensive characterization of MP dynamics and its ecological and clinical context in hospitalized children. Hubei Province, where the study was conducted, is representative of central China in terms of its temperate–subtropical monsoon climate, population density, and established MP epidemic patterns, with outbreaks typically following a 3–7 year cycle across the region (2, 4, 7, 15). *Mycoplasma pneumoniae* exhibited a pronounced boom-and-bust cycle: activity remained low in 2022, surged to an unprecedented peak in 2023, and then declined to near-undetectable levels by 2025. This oscillation occurred alongside shifts in the circulation of other respiratory pathogens, with RSV, FluA, and HRV showing increased activity during periods of low MP circulation. Clinically, pneumonia risk among MP-infected children was highest during the 2023 peak, remained elevated in 2024, and returned to baseline by 2025. This demographic composition reflects the typical inpatient pediatric population of a provincial tertiary maternal and child health hospital in central China. Together, these findings delineate a complete post-pandemic epidemic cycle of MP and its interaction with the broader respiratory pathogen ecosystem.

The delayed resurgence of MP in 2023, followed by its rapid decline, may be explained by immunity debt, an expanded pool of immunologically naïve children after prolonged low pathogen exposure, and subsequent susceptible depletion, although these mechanisms were not directly measured in our study. During 2020–2022, minimal MP circulation likely allowed a cohort of immunologically naïve children, particularly those born after 2020, to accumulate, resulting in a larger susceptible pool than in pre-pandemic cycles (11–16). When NPIs were relaxed, MP, whose transmission is typically slower and more protracted than that of many respiratory viruses, appeared to rebound later than RSV and FluA, consistent with reports of staggered post-pandemic rebounds across pathogens (13, 14, 17). Once MP transmission re-established, this enlarged susceptible pool may have amplified the 2023 outbreak, reflected by the fitted peak positivity of 35.1% in September. The subsequent collapse to 0.4% in 2025 aligns with depletion of susceptible hosts, leaving insufficient immunity gaps to sustain ongoing transmission. The relatively stable median age across the study period suggests that this depletion occurred broadly across the pediatric population rather than being confined to a narrow age band, consistent with observations of widespread post-pandemic MP involvement across childhood. The near-disappearance of MP from 2020 to 2022 and its subsequent resurgence in 2023 coincided with the emergence and evolution of long COVID in pediatric populations. Prior SARS-CoV-2 infection and long COVID may have altered host immune responses, respiratory susceptibility, and healthcare-seeking behaviors, potentially interacting with post-pandemic MP circulation patterns (18). Although individual-level COVID-19 history was not available in this dataset, the temporal dynamics observed here should be interpreted with consideration of the broader post-pandemic epidemiological context, including the potential role of long COVID in shaping population immunity and clinical presentations.

The *z*-score–standardized activity profiles and correlation matrix indicate that the MP boom-and-bust cycle unfolded within a dynamically reconfiguring pathogen landscape. During the 2023 MP peak, several viral pathogens, particularly RSV and FluA, showed reduced activity, consistent with their negative correlations with MP and reports of asynchronous post-NPI rebounds (19, 20). In contrast, during 2024–2025, as MP activity declined, RSV, FluA, and HRV exhibited heightened circulation, with RSV reaching 13.4% positivity in 2025, the highest among all pathogens that year. This pattern is compatible with ecological niche replacement, in which periods of attenuated MP circulation coincide with increased activity of viral pathogens occupying overlapping transmission spaces (19, 20). Positive correlations between MP and HAdV or hMPV may reflect partially shared seasonal windows or co-facilitating contact structures, while the strong HRV–HPIV correlation suggests closely aligned epidemiological niches and potentially similar responses to changes in population behavior and NPIs (21–23). Taken together, these findings underscore that MP dynamics cannot be interpreted in isolation but must be viewed within a multi-pathogen framework to understand post-pandemic respiratory infection patterns (19–23).

Marked variation in pneumonia risk across the MP cycle also carries important clinical implications. The three-fold increase in pneumonia odds in 2023 compared with 2022 indicates that the resurgence wave was associated with greater clinical severity. This pattern aligns with post-COVID-19 observations of increased MP disease burden and more severe presentations in children (16, 24). Potential contributors include infection of previously unexposed cohorts, higher epidemic intensity, or changes in circulating MP strains or macrolide resistance, although the present study could not directly assess these factors (25, 26). The persistently elevated pneumonia risk in 2024, despite declining MP activity, suggests that residual susceptibility or delayed healthcare-seeking behavior may have prolonged the period of increased severity. By 2025, pneumonia risk returned to the low levels observed in 2022, paralleling the near-disappearance of MP circulation. This complete temporal trajectory, from early-post-pandemic low levels to the 2023 peak and back to low levels in 2025, underscores the need for heightened clinical vigilance during and immediately after MP resurgence waves, as periods of increased pneumonia risk may extend beyond the peak in case numbers.

This study has several strengths. The four-year uninterrupted surveillance period captured the full post-pandemic MP cycle from resurgence to collapse. The use of a standardized multiplex PCR panel enabled simultaneous assessment of MP and 10 other respiratory pathogens, providing an integrated ecological perspective. Methodologically, the combination of offset-adjusted generalized additive models, *z*-score normalization, and multivariable logistic regression allowed robust quantification of temporal, ecological, and clinical patterns.

Several limitations should be acknowledged. First, as this was a single-center study, the epidemiologic and clinical patterns observed here may not fully generalize to settings with different healthcare-seeking behaviors, testing strategies, or post-pandemic respiratory pathogen dynamics. Second, although analyses adjusted for age, sex, and co-infection, residual confounding from unmeasured factors cannot be excluded, including the absence of important covariates such as vaccination status. In addition, detailed individual-level data on comorbidities, complications, and chronic conditions were not available, which may lead to unmeasured confounding and limit interpretation of clinical severity patterns. Third, the decline in testing volume over time may have influenced the observed trends, although monthly testing volume was incorporated as an offset in the regression models to partially mitigate this bias. Fourth, pneumonia diagnosis relied on clinical and radiographic assessment, which may introduce variability across clinicians and over time. Fifth, the small number of MP-positive cases in 2025 resulted in wide confidence intervals for that year’s pneumonia risk estimate, and limited sample sizes in certain age strata constrained the depth of age-stratified analyses and may have reduced the precision of age-specific estimates. Finally, the correlation matrix was based on monthly aggregated data and cannot be used to infer individual-level co-infection risk. Furthermore, the ecological correlations presented here reflect population-level temporal co-variation and do not imply causality. Moreover, strain- or genotype-level data were not available, preventing assessment of whether pathogen evolution or genotype-specific factors contributed to the observed epidemic dynamics. Although immunity debt and susceptible depletion are discussed as plausible explanatory frameworks, population immunity and susceptibility were not directly assessed, and these mechanisms should therefore be interpreted as hypotheses rather than proven causal pathways.

The near-disappearance of MP in 2025 also raises an important forward-looking question: when will the next resurgence occur? Based on the susceptible-accumulation framework, the timing of future epidemics will depend on how rapidly new susceptible cohorts, particularly children born after 2025, enter the population, as well as the pace at which immunity wanes among those infected during the 2023–2024 wave. Continuous multi-pathogen surveillance over the coming years will therefore be essential to detect the earliest signals of MP’s return and to distinguish true resurgence from the routine seasonal oscillations of viral respiratory pathogens. Recent advances in non-invasive diagnostic and prognostic tools for pediatric respiratory infections have shown promise in improving early risk stratification and reducing procedural burden (27–29). Although such tools were not available during our study period, integrating non-invasive approaches into future multi-pathogen surveillance frameworks may enhance early identification of high-risk children and complement laboratory-based pathogen detection.

In conclusion, this study documents a complete post-pandemic epidemic cycle of MP in hospitalized children in Hubei Province. The observed pattern is compatible with immunity-debt and susceptible-depletion hypotheses, although these mechanisms were not directly assessed. The cycle unfolded within a dynamically reconfiguring respiratory pathogen ecosystem, with RSV, FluA, and HRV showing increased activity as MP circulation waned. Clinically, the 2023 resurgence wave was associated with substantially increased pneumonia risk, which persisted into 2024 before returning to the early-post-pandemic low levels observed in 2022. These findings underscore the importance of multi-pathogen surveillance, preparedness for heightened clinical severity during MP resurgence, and recognition that large MP outbreaks may be self-limiting once the susceptible pool is exhausted. As respiratory pathogen dynamics continue to stabilize in the post-pandemic era, integrating MP monitoring into broader surveillance platforms will be essential for early detection of future resurgence. Together, these results highlight the need for sustained vigilance and public health readiness as respiratory pathogen ecosystems evolve beyond the COVID-19 period.

## Data Availability

The original contributions presented in the study are included in the article/Supplementary material, further inquiries can be directed to the corresponding author.
