# Supplementary material for: Post-pandemic resurgence and collapse of Mycoplasma pneumoniae in hospitalized children: a four-year multiplex surveillance study in China
Source: Front Public Health. 2026 Jun 4;14:1833309. doi: 10.3389/fpubh.2026.1833309 (PMC13275386; doi:10.3389/fpubh.2026.1833309)

Supplementary Table S1. Sensitivity analysis of *Mycoplasma pneumoniae* activity using negative binomial regression with half‑year periods, 2022–2025.

| **Time period** | **IRR** | **95% CI** |
| --- | --- | --- |
| 2023H1 (ref) | 1 | – |
| 2022H1 | 1 | 0.82–1.23 |
| 2022H2 | 2.34 | 1.94–2.83 |
| 2023H2 | 7.64 | 6.46–9.11 |
| 2024H1 | 2.2 | 1.71–2.81 |
| 2024H2 | 1.64 | 1.32–2.04 |
| 2025H1 | 0.085 | 0.038–0.161 |
| 2025H2 | 0.135 | 0.053–0.279 |

Note: IRR = Incidence Rate Ratio. H1 = January–June (first half of the year); H2 = July–December (second half of the year). The reference category is 2023H1. IRR < 1 indicates significantly fewer cases compared with 2023H1, while IRR > 1 indicates significantly more cases.

Supplementary Figure 1. Monthly *Mycoplasma pneumoniae* positivity and testing volume, 2022–2025.


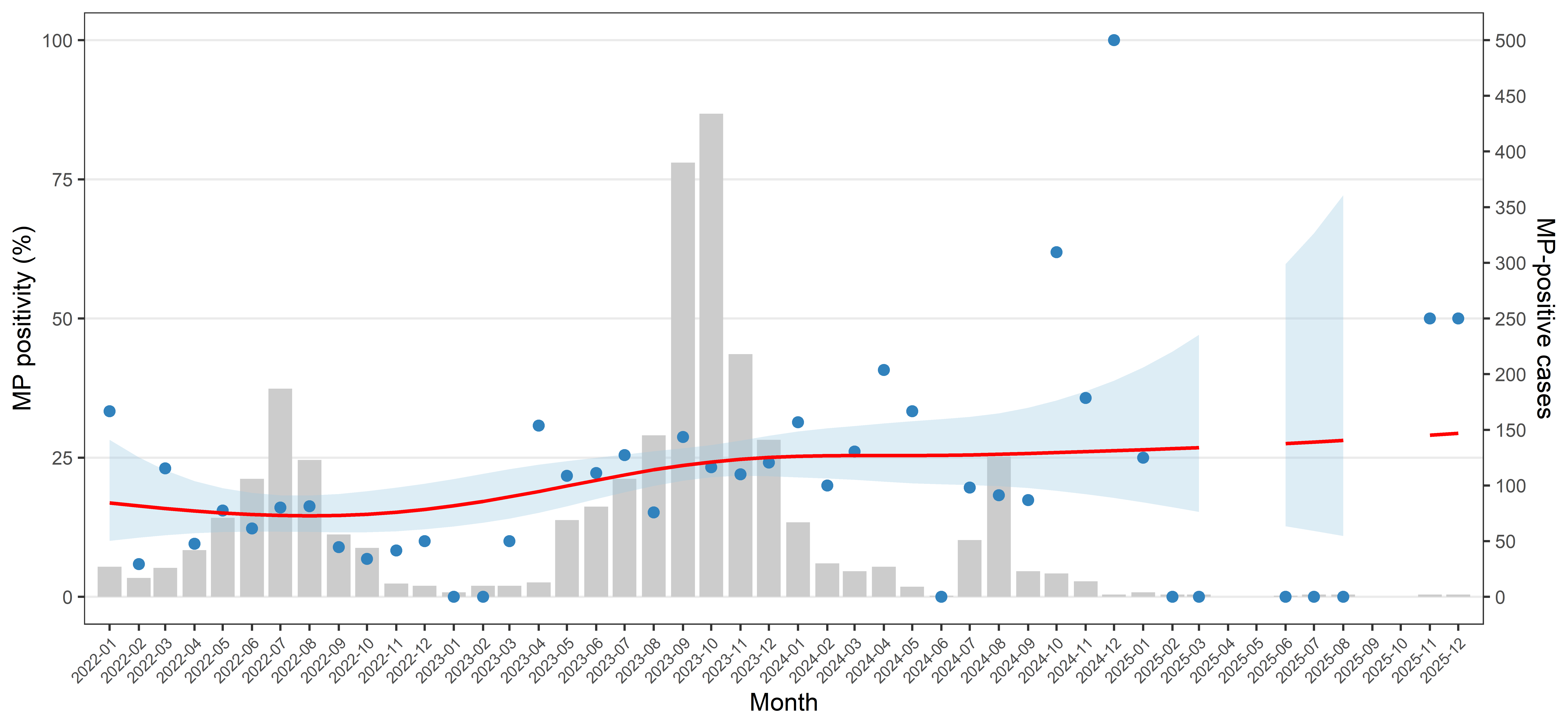


Supplementary Figure 2. Spearman correlation matrix of monthly positivity for respiratory pathogens, 2022–2025.


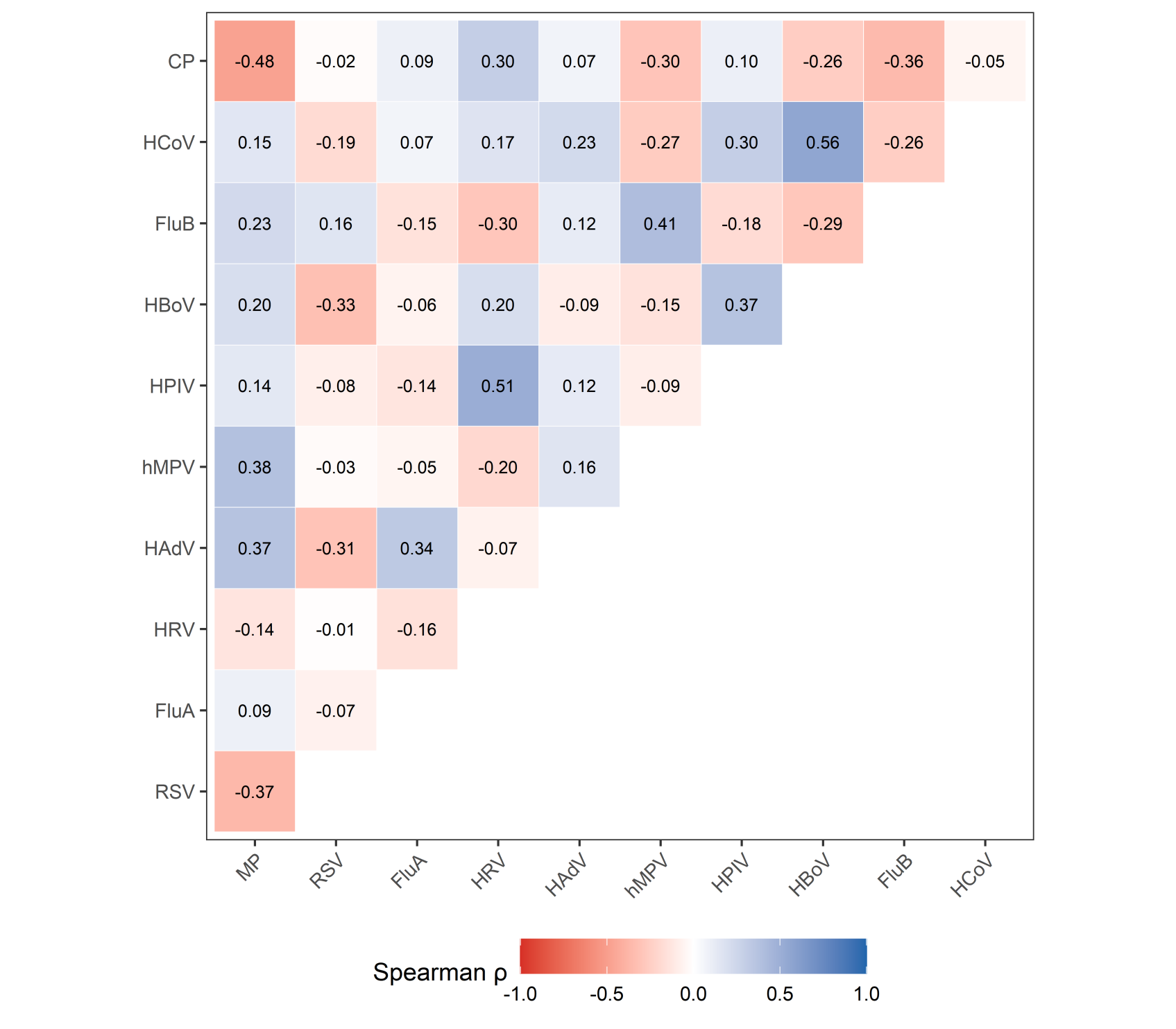

Supplement: Supplementary file 1 [file Table_1.DOCX]
